# Reference Genes for Expression Analyses by qRT-PCR in *Phthorimaea operculella* (Lepidoptera: Gelechiidae)

**DOI:** 10.3390/insects13020140

**Published:** 2022-01-28

**Authors:** Chen-Hui Shen, Li-Juan Peng, Yu-Xing Zhang, Hua-Rui Zeng, Hong-Fei Yu, Lin Jin, Guo-Qing Li

**Affiliations:** Education Ministry Key Laboratory of Integrated Management of Crop Diseases and Pests, State & Local Joint Engineering Research Center of Green Pesticide Invention and Application, Department of Entomology, College of Plant Protection, Nanjing Agricultural University, Nanjing 210095, China; 2019202031@njau.edu.cn (C.-H.S.); 2021802177@stu.njau.edu.cn (L.-J.P.); 2020202042@stu.njau.edu.cn (Y.-X.Z.); 2021102074@stu.njau.edu.cn (H.-R.Z.); 2021802180@stu.njau.edu.cn (H.-F.Y.); ligq@njau.edu.cn (G.-Q.L.)

**Keywords:** *Phthorimaea operculella*, reference gene, ribosomal protein, elongation factor, chitin synthase

## Abstract

**Simple Summary:**

Quantitative real-time fluorescent polymerase chain reaction (qRT-PCR) is a momentous tool for calculating the expression levels of targeted genes across various experimental conditions. The selection and evaluation of stable reference genes for qRT-PCR analysis is an essential precondition for reliable expression assessment. *Phthorimaea operculella* is one of the most serious Lepidopteran pests that attack potatoes around the world. In the present paper, a total of 10 commonly used reference genes, namely *ACT*, *α-TUB*, *18S*, *28S*, *GAPDH*, *EF1α*, *RPL4*, *RPL13*, *RPL27* and *SOD*, were selected and validated for suitability under three treatments (developmental stages, tissues/organs and temperatures) using five methods (Ct value, geNorm, NormFinder, BestKeeper and RefFinder). These results indicated that *EF1α* and *RPL13* were the best suitable reference genes for diverse backgrounds. The relative transcript levels of the target gene *chitin synthase A* gene (*PoChSA*) were abundantly expressed in epidermal cells, and lowly transcribed in the midgut. Our findings will be beneficial for improving the accuracy of qRT-PCR analysis for future functional analysis of the target gene expression in *P. operculella*.

**Abstract:**

Due to a lack of effective internal references, studies on functional genes in *Phthorimaea operculella*, a serious Lepidopteran pest attacking potatoes worldwide, have been greatly limited. To select suitable endogenous controls, ten housekeeping genes of actin (*ACT*), α-tubulin (*α-TUB*), glyceraldehyde-3-phosphate dehydrogenase (*GAPDH*), elongation factor 1α (*EF1α*), *18S* and *28S* ribosomal RNA (*18S*, *28S*), ribosomal protein genes *RPL4*, *RPL13* and *RPL27* and superoxide dismutase (*SOD*) were tested. Their expression levels were determined under three different experimental conditions (developmental stages, tissues/organs and temperatures) using qRT-PCR technology. The stability was evaluated with five methods (Ct value, geNorm, NormFinder, BestKeeper and RefFinder). The results clarified that *RPL13*, *EF1α* and *RPL27* are ranked as the best reference gene combination for measuring gene expression levels among different developing stages and under various temperatures; *EF1α* and *RPL13* are recommended to normalize the gene expression levels among diverse tissues. *EF1α* and *RPL13* are the best reference genes in all the experimental conditions. To validate the utility of the selected reference pair, *EF1α* and *RPL13*, we estimated the tissue-biased expression level of chitin synthase A gene (*PoChSA*). As expected, *PoChSA* was abundantly expressed in ectodermally derived epidermal cells, and lowly transcribed in the midgut. These findings will lay the foundation for future research on the molecular physiology and biochemistry of *P. operculella*.

## 1. Introduction

Quantitative real-time fluorescent polymerase chain reaction (qRT-PCR) is a powerful tool for the quantification of nucleic acids owing to its advantages of high specificity, sensitivity, accuracy and rapidity [1,2]. It has been widely used in scientific research [3,4]. When qRT-PCR is used to calculate the relative expression levels of target genes, it is necessary to combine relatively stable reference genes for normalization to improve the quantitative results [5]. If unsuitable references are applied, the nucleic acid quantitates will be biased. In *Locusta migratoria*, for instance, inappropriate selection of the reference genes results in significant differences in the expression level of the target gene chitin synthase 1 [6]. Therefore, stably expressed reference genes should be selected under different treatments, within different tissues or organs and at different developmental stages [7,8].

In general, the reference genes are housekeeping genes (HKGs) that stably transcribe in various cells or during diverse physiological states [9]. However, there is no single universal reference gene [10]. To obtain accurate results, the exact experimental conditions for the expression of each candidate reference gene must be verified [7,8].

The potato tuber moth, *Phthorimaea operculella* (Lepidoptera, Gelechiidae), is one of the most serious Lepidopteran pests attacking potatoes around the world [11]. It reduces potato production either via mining and damaging leaves and stems in fields or via burrowing and destroying tubers in storage [4]. In *P. operculella*, the ACTIN (*ACT*) gene is used for qRT-PCR studies when measuring the expression of two pheromone receptor genes *OR1* and *OR3* [4] and the level of chitin synthase A genes [3]. However, the stability and effectiveness of *ACT* have not been validated. This might significantly affect statistical analyses and might result in false data interpretation [12]. Therefore, it is imperative to identify the optimal endogenous controls for specific conditions in *P. operculella*.

The stability of reference genes has widely been evaluated in Lepidopterans [7,13,14,15,16,17,18,19,20]. For instance, the most suitable reference genes have been documented in *Tuta absoluta* (elongation factor 1α, *EF1α*; *60S* ribosomal protein L28, *RPL28*) [7], *Thitarodes armilicanus* (glyceraldehyde-3-phosphate dehydrogenase, *GAPDH*) [21], *Diaphania caesalis* (*ACT* and 60S ribosomal protein *RPL13a* across developing stages, *ACT* and eukaryotic initiation factor *EIF4A* in various tissues) [22] and *Sesamia inferens* (18S ribosomal RNA, *18S*; ribosomal protein *S20*, *RPS20*; α-tubulin, *α-TUB*) [23]. Generally, at least two reference genes may be necessary for each insect species as a single reference gene cannot satisfy all experimental requirements [24].

Since the top 10 most frequently used reference genes include *ACT*, *RPL*, *TUB*, *GAPDH*, *RPS*, *18S*, *EF1α*, *TATA*, *HSP* and *SDHA* in insects [25], we accordingly selected ten HKGs, i.e., *ACT*, *α-TUB*, *18S*, *28S*, *GAPDH*, *EF1α*, *RPL4*, *RPL13*, *RPL27* and *SOD*, in *P. operculella*. The objectives of this survey were to (i) evaluate the expression stability of the 10 candidate reference genes, (ii) screen/select the most stable internal reference genes expressed in different developing stages and tissues/organs and under different temperatures and (iii) to validate the stability and effectiveness of the selected reference gene pair by comparison with the published results. Our results provide the reference basis for further molecular studies involving *P. operculella*.

## 2. Materials and Methods

### 2.1. Insects

*P. operculella* used for this study were collected from *Solanum melongena* L. in Guiyang city, Guizhou Province, China in 2020. The larvae were routinely maintained in an insectary at 26 ± 1 °C under a 12 h:12 h light-dark photoperiod and 60–80% relative humidity using fresh potatoes as food. The adults were fed with a 10% honey solution.

### 2.2. Samples through Developing Stages

All stages of *P. operculella* were sampled: young and old larvae, pupae and adults. The number of individuals for each replicate across the different developmental stage was as follows: 10 young larvae, 5 old larvae, 5 pupae and 5 adults (3 males and 2 females). The collection was repeated three times.

### 2.3. Specimens among Various Tissues

Ten fully grown larvae were selected as a replicate. They were dissected and the head capsule, foregut, midgut, hindgut, fat body, hemocytes and epidermis were collected. The tissue collection was repeated three times. The tissue specimens were placed in RNAlater R (Thermo Fisher Scientific Inc., Waltham, MA, USA) and stored for several weeks at −80 °C before total RNA isolation.

### 2.4. Collections during Varied Temperature Incubation

The final instar larvae were transferred into three temperatures (4 °C, 26 °C and 35 °C). Ten larvae as a replicate were collected after 2, 6 and 12 h. A total of nine treatments were set. The collection was repeated three times and stored for several weeks at −80 °C before total RNA isolation.

### 2.5. Samples for the Expression Analysis of PoChSA

The ultimate instar larvae were dissected and the head capsule, foregut, midgut, hind gut and epidermis were collected. A total of 10 individuals were dissected for each replicate. The tissue collection was repeated three times. The tissue specimens were placed in RNAlater R (Thermo Fisher Scientific Inc., Waltham, MA, USA) and stored for several weeks at −80 °C before total RNA isolation.

### 2.6. Selection and Authentication of Candidate HKGs

Ten HKG sequences (actin, *ACT*; α-tubulin, *α-TUB*; glyceraldehyde-3-phosphate dehydrogenase, *GAPDH*; elongation factor 1α, *EF1α*; *18S* and *28S* ribosomal RNA, *18S* and *28S*; ribosomal proteins *RPL4, RPL13* and *RPL27*; superoxide dismutase, *SOD*) were selected. The accession numbers of these genes are listed in Table 1.

Reverse transcription PCR (RT-PCR) was performed to authenticate the HKGs using the primers listed in Table 1. The amplified products were separated by electrophoresis on 1.5% agarose gel and purified using the Wizard^®^ PCR Preps DNA Purification System (Promega, Madison, WI, USA). Purified DNA was ligated into the pGEM^®^-T easy vector (Promega, Madison, WI, USA) and several independent subclones were sequenced from both directions. The resultant sequences were submitted to GenBank; the accession numbers are listed in Table 1.

### 2.7. Quantitative Real-Time PCR (qRT-PCR)

The qRT-PCR primers were designed using Beacon Designer 7 (Premier Biosoft International, Palo Alto, Santa Clara, CA, USA), and are given in Table 2. The qRT-PCR reactions were performed using ChamQ Universal SYBR qPCR Master Mix (Vazyme Biotech Co., Ltd., Nanjing, China) and QuantStudio™ 7 Pro Real-Time PCR System (Applied Biosystems, Life Technologies, Carlsbad, CA, USA) according to the manufacturer’s protocol. The reaction mixture consisted of 1 μL of cDNA template, 10 μL of 2× ChamQ Universal SYBR qPCR Master Mix, 0.4 μL of forward primer (10 μM), 0.4 μL of reverse primer (10 μM) in a final reaction volume of 20 μL. A reverse transcription negative control (without reverse transcriptase) and a non-template negative control were included for each primer set to confirm the absence of genomic DNA and to check for primer dimers or contamination in the reactions, respectively. The qRT-PCR protocol included an initial step of 95 °C for 30 s, followed by 40 cycles of 95 °C for 5 s and then annealed at 60 °C for 34 s, followed by one cycle of 95 °C for 15 s, 60 °C for 60 s and 95 °C for 1 s. PCR amplicons were subjected to melting curve analysis. The specificity of the qRT-PCR reactions was monitored by melting curve analysis using QuantStudio™ Design & Analysis Software (version 1.5.0) and gel electrophoresis. Amplification efficiencies were determined by a 10-fold dilution series of template. All experiments were repeated in triplicate.

### 2.8. Evaluation of Reference Gene Selection

*ChSA* of *P. operculella* was used to evaluate the stability of candidate reference genes. The primer sequence of the target gene was as follows: forward (5′-GCCTGGAGTTCACAGTCAGA-3′) and reverse (5′-GCCGGTCTTTCTTAAGTTGC-3′). The average relative levels of *PoChSA* in different tissues were computed based on 2^−ΔΔCT^ method and from three replicates. We used SPSS for Windows (Chicago, IL, USA) for statistical analyses. The averages (±SE) were submitted to analysis of variance with the Tukey–Kramer test.

### 2.9. Data Processing

The raw Ct values were obtained using the QuantStudio™ Design & Analysis Software (version 1.5.0). The algorithms, including geNorm [26], BestKeeper [27] and Normfinder [28], were used to analyze the stability of selected HKGs, strictly following the manuals of the algorithms. Finally, the comprehensive ranking of each condition was obtained according to RefFinder [29,30].

## 3. Results

### 3.1. Selection of Candidate HKGs

We selected ten HKG genes and designated them as *ACT*, *α-TUB*, *18S*, *28S*, *GAPDH*, *EF1α*, *RPL4*, *RPL13*, *RPL27* and *SOD*. The resultant sequences were submitted to GenBank; the accession numbers were listed in Table 1. The correctness of the ten HKGs was proven by RT-PCR.

The products from qRT-PCR were confirmed by sequencing. The primer specificity for qRT-PCR was verified by melting curve analysis. All the primer pairs amplified a single PCR product with the expected sizes and sequences, showed a slope less than −3.0 and exhibited regression coefficient (R2) and efficacy values ranging from 0.991–0.999 and 93.80–103.89% (Table 2). These data indicate that the amplification efficiencies of the primers reached the standard requirements of conventional qRT-PCR [5].

### 3.2. Expression Variations of the Ten HKGs

The specimens were collected from four developmental stages (young and old larvae, pupae and adults), seven larval tissues (head capsule, foregut, midgut, hindgut, fat body, hemocytes and epidermis) and three temperature treatments (4 °C, 26 °C and 35 °C). Using the products obtained by qRT-PCR for agarose gel electrophoresis, we found that all ten genes had single amplicons of expected size (data not shown). Therefore, these ten genes were expressed during different developmental stages, among different larval tissues and under different temperatures.

**Figure 1:**
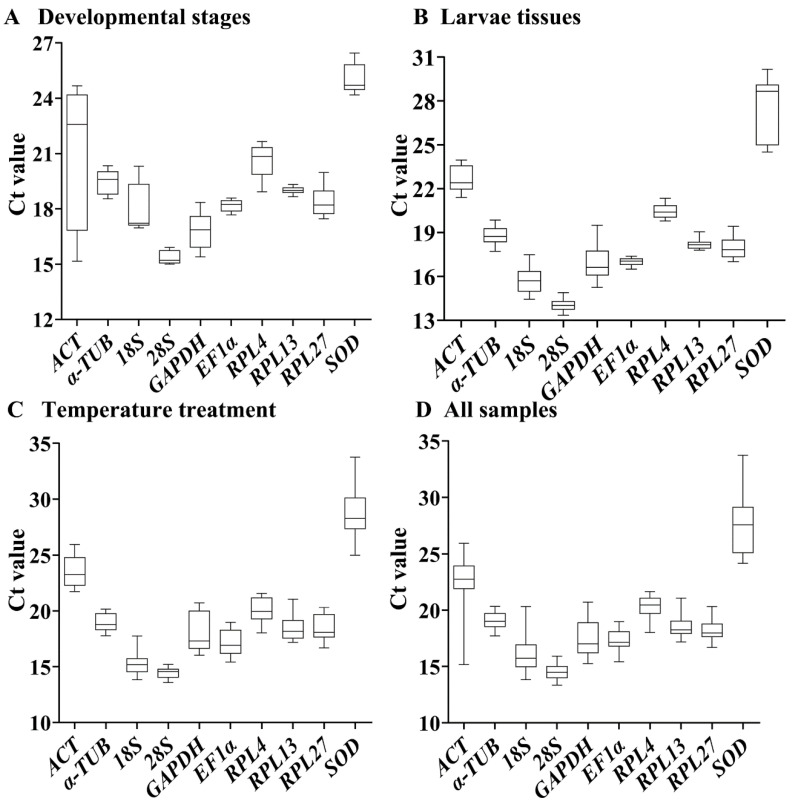
Expression levels of ten house-keeping genes in *Phthorimaea operculella*. The mean Ct values for 10 candidate reference genes are shown in three independent experiments: developmental stage, tissue, and temperature. Each box indicates the 25th and 75th percentiles. The line across the box represents the median. Abbreviation: ACT, actin; α-TUB, α-tubulin; GAPDH, glyceraldehyde-3-phosphate dehydrogenase; EF1α, elongation factor 1α; 18S and 28S, 18S and 28S ribosomal RNA; RPL4, RPL13 and RPL27, ribosomal protein; SOD, superoxide dismutase. The abbreviations are exactly the same as Figure 2, Figure 3, Figure 4 and Figure 5.

The overall threshold cycle (Ct) values under different experimental conditions are shown in Figure 1 and Table S1. Across developing stages, *EF1α* and *RPL13* had the smaller gene expression variation, whereas *ACT* and *18S* had the higher expression difference (Figure 1A). Among various tissues, except for *GAPDH* and *SOD*, the expression fluctuations were small in selected HKGs (Figure 1B). Under different temperatures, the expression fluctuations were small in selected HKGs except for *SOD* and *GAPDH* (Figure 1C). A combination of these results revealed that the variations in *RPL13*, *EF1α*, *RPL27* and *α-TUB* were smaller, whereas the ranges in *ACT*, *GAPDH*, *18S* and *SOD* were larger (Figure 1D).

### 3.3. Expression Stability of the Ten HKGs during Developmental Stages

**Figure 2:**
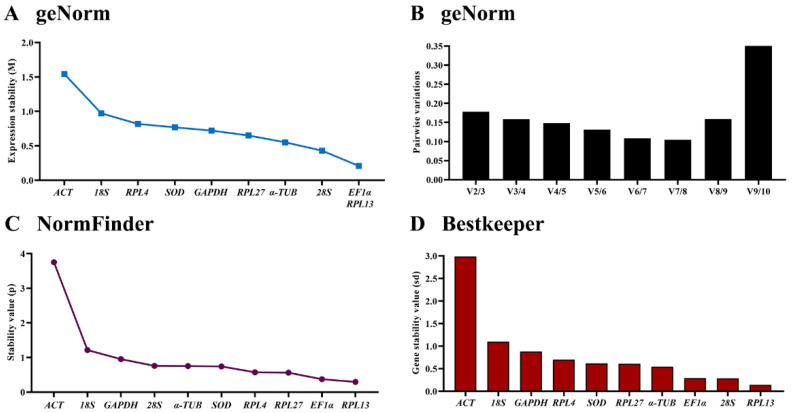
Expression stability of ten house-keeping genes during development stage in *Phthorimaea operculella*. All stages of P. operculella were sampled: young and old larvae, pupae and adults (collected on the first and second days of each stage). The expression stability rankings were determined by geNorm, NormFinder and BestKeeper.

The geNorm algorithm evaluates the candidate reference genes based on their expression stability values (M-values) and pairwise variations (Vn/Vn+1). The expression stability values revealed that *EF1α*, *RPL13* and *28S* were the better reference genes during developing, with M-values below 0.5. The values of other genes were below 1, except for *ACT*, and their stability values were similar (Figure 2A, Table 3). The pairwise variation analysis showed that the V3/4 value was near 0.15; indicating three different reference genes are needed for gene expression analysis during development (Figure 2B).

According to the NormFinder, those genes with low stability values, based on intra- and inter-group expression variations, are considered to be the most stable reference genes. Across different development stages, the stable genes were *RPL13*, *EF1α* and *RPL27*, with the *p* value less than 1.0. The most unstable gene was *ACT*, with the *p* value of 3.8 (Figure 2C, Table 3).

Based on the BestKeeper analysis, the stable orders of selected HKGs were *RPL13*, *28S*, *EF1α*, *α-TUB*, *RPL27*, *SOD*, *RPL4*, *GAPDH*, *18S* and *ACT*, from the most stable to the least. The last two genes, *18S* and *ACT*, had Cp values of more than 1 (Figure 2D, Table 3), indicating that they should be excluded as reference genes for qRT-PCR to test the expression level of the target gene.

The online tool RefFinder combines the three methods above to compare and rank the tested reference genes [29]. It ranks the selected HKGs in the following order from the most to least stable: *RPL13* > *EF1α* > *RPL27* > *28S* > *α-TUB* > *RPL4* > *SOD* > *GAPDH* > *18S* > *ACT* (Figure 5A). Therefore, *RPL13*, *EF1α* and *RPL27* are ranked as the best reference gene combination for measuring target genes among different developing stages.

### 3.4. Expression Stability of the Ten HKGs among Different Tissues

**Figure 3:**
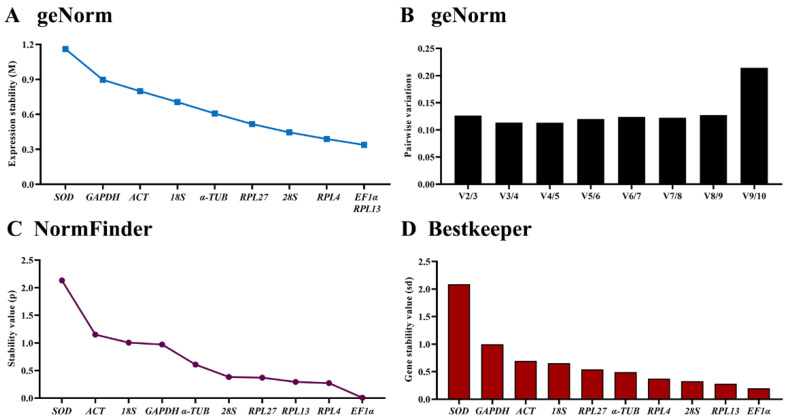
Expression stability of ten house-keeping genes among various tissues in *Phthorimaea operculella*. Head capsule, foregut, midgut, hindgut, fat body, hemocytes and epidermis were dissected from the fourth instar larvae. The expression stability rankings were determined by geNorm, NormFinder and BestKeeper.

Among the three tissues, the stability of the selected HKGs were *EF1α* = *RPL13* > *RPL4* > *28S* > *RPL27* > *α-TUB* > *18S* > *ACT* > *GAPDH* > *SOD*; based on the geNorm algorithm, the M-values of *EF1α*, *RPL4* and *RPL13* were below 0.4 (Figure 3A, Table 3). The pairwise variation analysis displayed that the V2/3 to V8/9 values were below 0.15, suggesting two reference genes are enough for gene expression determination within various tissues (Figure 3B). 

The NormFinder analysis revealed that the stability of the selected HKGs were *EF1α* > *RPL4* > *RPL13* > *RPL27* > *28S* > *α-TUB* > *GAPDH* > *18S* > *ACT* > *SOD*, with the *p* value of 0.007, 0.272, 0.293, 0.370, 0.383, 0.608, 0.972, 1.004, 1.150 and 2.131, respectively. Again, the *p* values of *EF1α*, *RPL4*, *RPL13*, *RPL27* and *28S* were below 0.4 (Figure 3C, Table 3), indicating their similar stability.

The BestKeeper data uncovered that *EF1α*, *RPL13*, *28S*, *RPL4* were the most stable because they showed Cp values of 0.200, 0.279, 0.328 and 0.373, respectively. The Cp values of *α-TUB*, *RPL27*, *18S*, *ACT* and *GAPDH* were less than 1.0., and the Cp value of *SOD* was more than 2.0 (Figure 3D, Table 3). 

The RefFinder showed a comprehensive ranking order from the most to the least stable: *EF1α* > *RPL13* > *RPL4* > *28S* > *RPL27* > *α-TUB* > *18S* > *GAPDH* > *ACT* > *SOD* (Figure 5B). Thus, the two HKGs (*EF1α* and *RPL13*) are recommended to be used to test the target gene expression levels among various tissues.

### 3.5. Stability of the Ten HKGs under Different Temperatures

**Figure 4:**
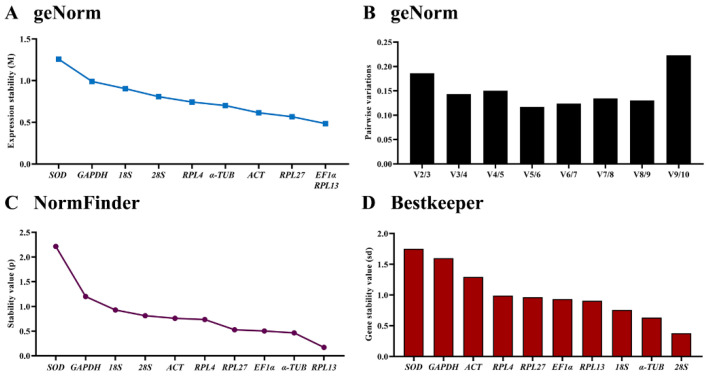
Expression stability of ten house-keeping genes under different temperatures in *Phthorimaea operculella*. The fourth-instar larvae reared under three temperatures (4 °C, 26 °C and 35 °C) were collected. The expression stability rankings were determined by geNorm, NormFinder and BestKeeper.

The geNorm algorithm results showed that the comprehensive reference gene rankings from the best to the least stable were *EF1α*, *RPL13*, *RPL27*, *ACT*, *α-TUB*, *RPL4*, *28S*, *18S*, *GAPDH* and *SOD* (Figure 4A, Table 3). Except for *SOD*, the other genes in the selected HKGs showed values below 1, indicating their stabilities were similar. Moreover, the pairwise variation analysis showed that the V3/4 value was below 0.15, indicating three different reference genes are needed for gene expression analysis under different temperatures (Figure 4B).

By the NormFinder analysis, the stable orders of the selected HKGs from the most stable to the least were *RPL13*, *α-TUB*, *EF1α*, *RPL27*, *RPL4*, *ACT*, *28S*, *18S*, *GAPDH* and *SOD*. Again, the *p* values revealed by the NormFinder analysis indicated that *RPL13*, α-*TUB*, *EF1α*, *RPL27* were smaller, demonstrating that the genes have similar stability (Figure 4C, Table 3).

The BestKeeper data unveiled that the steady orders were *28S*, *α-TUB*, *18S*, *RPL13*, *EF1α*, *RPL27* and *RPL4* (Figure 4D, Table 3). Since the Cp values of *ACT*, *GAPDH* and *SOD* were more than 1, they cannot be used as reference genes for qRT-PCR to test the expression level of the target gene. The other genes showed values below 1, indicating their stability values were similar (Figure 4D, Table 3).

**Figure 5:**
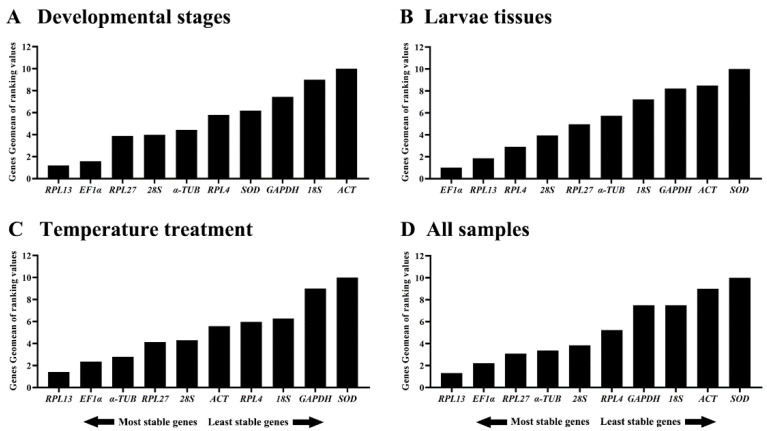
Expression stability of ten house-keeping genes in different samples of *Phthorimaea operculella*. The stability of the reference genes calculated by the Geomean method of RefFinder. A lower Geomean of ranking value denotes more stable expression.

According to the RefFinder results, the stability rankings were as follows: *RPL13* > *EF1α* > *α-TUB* > *RPL27* > *28S* > *ACT* > *RPL4* > *18S* > *GAPDH* > *SOD* (Figure 5C). 

When the three different conditions were combined together, the RefFinder results indicated that the stability rankings from the most to the least were *RPL13*, *EF1α*, *RPL27*, *α-TUB*, *28S*, *RPL4*, *GAPDH*, *18S*, *ACT* and *SOD* (Figure 5D). Thus, the two HKGs (*EF1α* and *RPL13*) can be selected as reference genes for measuring the target gene expression levels among diverse backgrounds.

### 3.6. Validation of the Selected Reference Genes after Gene Expression

**Figure 6:**
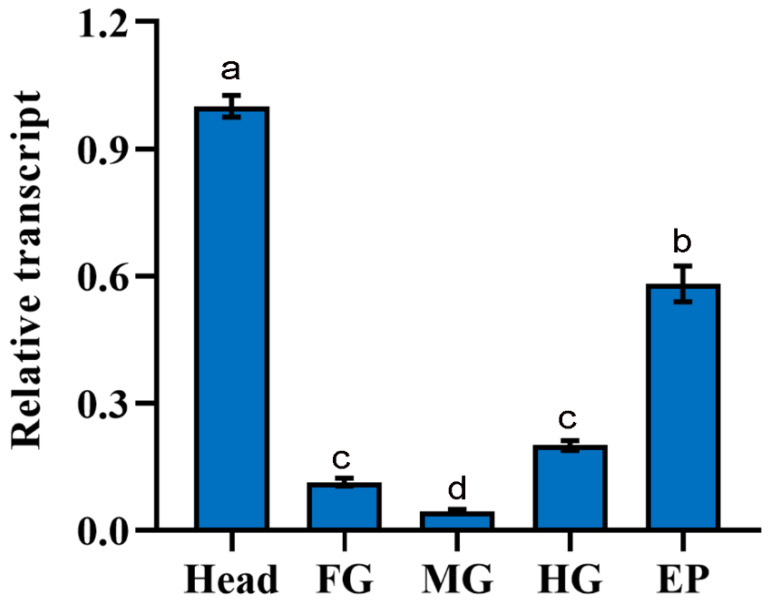
Tissue expression of the chitin synthase A gene (*PoChSA*) in *Phthorimaea operculella*. The head capsule (Head), foregut (FG), midgut (MG), hindgut (HG) and epidermis (EP) were dissected from the fourth-instar larvae. For each sample, 3 independent pools of 20–30 individuals were measured in technical triplicate using qRT-PCR. The values were calculated using the 2^−ΔΔCT^ method, using the selected reference genes *EF1α* and *RPL13*. The relative transcripts are the ratios of copy numbers in different developing stages relative to the head capsule, which is set as 1. The columns represent averages, with vertical lines indicating SE. Different letters indicate significant difference at *p* value < 0.05 using analysis of variance with the Tukey–Kramer test.

To demonstrate the utility of *EF1α* and *RPL13* in accurate gene expression analysis, the expressions of chitin synthase A gene (*PoChSA*) in the head capsules, epidermis, foregut, midgut and hindgut were calculated after normalization with a combination of *EF1α* and *RPL13*. The highest accumulated mRNA level of *PoChSA* was found in the head capsule and epidermis, followed by those in the foregut and hindgut; the lowest level was detected in the midgut (Figure 6).

## 4. Discussion

In the present paper, we investigated the expression stability of ten HKGs in *P. operculella*. Out of the ten HKGs, *ACT*, *RPL*, *TUB*, *GAPDH*, *18S* and *EF1α* are the top 10 most frequently used reference genes [25]. 

It is widely accepted that moderately expressed HKGs should be chosen as potential reference genes because genes with extremely high or low expression levels are less reliable [31]. According to the Ct value obtained in the present paper, *ACT* and *SOD* are the less expressed and *GAPDH* and *18S* are the most expressed. Even though the results obtained using the BestKeeper, geNorm and NormFinder algorithms were not completely consistent, the data still revealed that the mRNA levels of *ACT* and *SOD* are changed dramatically throughout the developing stages among tissues and under different temperatures, respectively. It appears that the four genes should be excluded as reference genes for qRT-PCR.

Actin (ACT) plays an important role in cell secretion, motility cytoplasm flow and experimental cytoskeleton maintenance and is abundantly expressed in most cell types. Even though *ACT* is used for qRT-PCR studies when measuring the expression of target genes in *P. operculella* [3,4], it was verified to be one of the most unstable genes in the present paper. The transcript level of *ACT* is also less stable in several Coleopteran insect species, such as *Phaedon brassicae*, *Henosepilachna vigintioctomaculata*, *Leptinotarsa decemlineata*, *Coleomegilla 11 aculate*, *Coccinella septempunctata* and *Hippodamia convergens* [32,33,34,35,36,37], although *ACT* is one of the most stable reference genes across several developmental stages in Orthopteran (*Schistocerca gregaria* and *Chortoicetes terminifera*), Hemipteran (*Diuraphis noxia*), Thysanopteran (*Thrips tabaci*), Hymenopteran (*Apis mellifera*), Dipteran (*Drosophila melanogaster* and *Liriomyza trifolii*) and Lepidopteran (*Plutella xylostella* and *Chilo suppressalis*) insects [13,14,15,16,17,18,19,20].

18S ribosomal RNA is a part of the ribosomal RNA (rRNA), which accounts for more than 80% of the total RNA pool [38], whereas mRNA accounts for only 3 to 5%. This is consistent with our data that *18S* is the most expressed in *P. operculella* (this study). Therefore, the use of rRNAs as reference genes may mask subtle changes in target mRNAs [39]. Moreover, *18S* shows a large variation in different development stages in *Myzus persicae* [40].

Superoxide dismutase (SOD) is known as an antioxidative stress protein by scavenging the superoxide radicals, used to defend against reactive oxygen species (ROS) damage caused by a variety of unfavorable environmental stressors in some insect species [41,42,43]. In this study, the *SOD* gene was verified to be one of the most unstable genes under three different conditions.

Similarly, the instability of *GAPDH* expression has been documented in *Colaphellus bowringi* [44], *D. caesalis* [22], *Mythimna separata* [45], *Ophraella communa* [46] and *P. brassicae* [37]. GAPDH functions as a glycolytic enzyme involved in glycolysis and is associated with cell proliferation under adverse conditions where its catalytic activity is impaired [47]. It is presumed that any perturbation toward energy metabolism or cell proliferation would have a potential impact on *GAPDH* expression. Considering these issues, it is inappropriate to adopt *GAPDH* as a reference gene. Therefore, we focus on five genes, i.e., *α-TUB*, *EF1α*, *RPL4*, *RPL13* and *RPL27*, in *P. operculella* for the selection of a suitable reference gene combination.

It has been suggested that multiple reference genes should be used in order to avoid biased normalization [48]. Additionally, from the present study, we recommended two reference genes, *EF1α* and *RPL13,* to normalize the gene expression levels among diverse conditions in *P. operculella*. Consistent with our results, the conserved nuclear gene elongation factor 1 alpha (EF1α) plays an important role in translation by catalyzing the GTP-dependent binding of aminoacyl-tRNA to the acceptor site of the ribosome. EF1α is evaluated as the most stable gene under diverse conditions in *D. melanogaster* [15], *C. terminifera* [13], *Bombus terrestris* and *Bombus lucorum* [49], *Frankliniella occidentalis* [50] and *Helicoverpa armigera* [39].

Ribosomal proteins are known to play an essential role in ribosome assembly, and they, in conjunction with four ribosomal RNAs (rRNAs), make up the ribosomal subunits responsible for cellular protein translation [51]. Similar to our results, ribosomal protein genes are the most widely selected reference genes for expression studies in insects during the past 10 years [25]. They are recommended as reference genes in Coleopteran species *P. brassicae* (*RPL32* and *RPL19*) [37], *Dendroctonus frontalis* (*RPS18*) [52], *H. vigintioctomaculata* (RPL13 and *RPS18*) [36], *L. decemlineata* (*RP18* and *RP4*) [32], *Lethrus apterus* (*RP18*) [53], *Mylabris cichorii* (*RPS22e*) [54] and *Tribolium castaneum* (*RPS3*, *RPL13a* and *RPL18*) [55,56], Hymenopterans such as *A. mellifera* (*RPS18*) [16] and *Aphidius gifuensis* (*RPL13*, *RPS18*, *RPL29*) [57], Lepidopterans such as *P. xylostella* (*RPS13* and *RPS23*) [58] and *H. armigera* (*RPS15* and *RPL27*) [39], Thysanopteran species *F. occidentalis* (*RPL32*) [50], Hemipteran species *Amrasca biguttula biguttula* (*RP13*) [59], *Aphis craccivora* (*RPL11*, *RPS8* and *RPL14*) [60], *Cimex lectularius* (*RPL18*) [61], *Lipaphis erysimi* (*RPL18* and *RPL13*) [62], *Phenacoccus solenopsis* (*RPL32*) [63], *Rhodnius prolixus* (*RPS18*) [64] and Orthopteran *S. gregaria* (*RP49*) [19], as well as Acari *Tetranychus cinnabarinus* (*RPS18* and *RP49*) [65] and *Tetranychus urticae* (*RP49*) [66].

To sum up, in this study, the genes *RPL13*, *EF1α* and *RPL27* are indicated to be ranked as the best reference gene combination for measuring gene expression levels among different developing stages and under various temperatures, while *EF1α* and *RPL13* are recommended to normalize gene expression levels among diverse tissues. *EF1α* and *RPL13* are the best reference genes in all the experimental conditions in *P. operculella* (Lepidoptera: Gelechiidae). Interestingly, in another Lepidoptera insect *Spodoptera frugiperda* (Noctuidae), based on the online program RefFinder, *SOD*, *RPL10* and *RPS24* were reported to be the most stable reference genes for different developmental stages, while *α-TUB*, *RPL10* and *ATP* were for various tissues, *AK*, *RPL10* and *18S* for mating status, *18S* and *AK* for hormone treatment, *18S*, *RPL10* and *SOD* for diets treatment, and *RPL10*, *18S* and *RPS24* for temperature treatment [67]. The results verified that the expression stability of the reference genes varied under different treatments. Similarly, the ribosomal protein genes are also the most stable reference genes selected under almost all the experimental conditions. In addition, the difference in housekeeping genes under similar treatments may be related to the phylogenetic relationship and feeding habits of the two lepidoptera insects.

In order to demonstrate the utility of *EF1α* and *RPL13* in accurate gene expression analysis in *P. operculella*, we evaluated the relative gene expression level of *PoChSA* in the head capsules, epidermis, foregut, midgut and hindgut. Our results showed that *PoChSA* was abundantly expressed in the head capsule and epidermis, moderately transcribed in the foregut and hindgut and lowly expressed in the midgut. Our expression data are consistent with the fact that *ChSA* encodes an enzyme that catalyzes the biosynthesis of chitin in the ectodermally derived epidermal cells forming epidermis, trachea, foregut and hindgut [3,68,69,70,71]. Thus, the tissue-biased expression pattern of *PoChSA* demonstrates that *EF1α* and *RPL13* can be used as endogenous controls to assess gene expression in *P. operculella*. 

## 5. Conclusions

Our findings recommend *EF1α* and *RPL13* as the optimal reference gene set under three different experimental conditions. *EF1α* and *RPL13* combinations can be proposed as reference genes for measuring the target gene expression levels among diverse backgrounds in *P. operculella*. To date, this is the first study to screen out candidate reference genes for gene expression analysis in *P. operculella*. The results lay a foundation for molecular research. Nevertheless, the application of these loci as reference genes under other physiological or experimental conditions remains to be determined.

## Figures and Tables

**Table 1:** A list of primers used for RT-PCR of the genes.

Gene Name	Primer Sequences (5′ to 3′)	Amplicon Size (bp)	Accession Number
*ACT*	Forward GTGTTCCCCTCCATCGTC Reverse ACATCGCCTGGAAAGTAG	979	OL675412
*α-TUB*	Forward GCCGTGTTTGTGGACTTG Reverse TGATGGAGGATACGATTTGA	523	OL690519
*18S*	Forward ATGCCCTTAGATGTCCTGG Reverse GGATTTCTAACCCGTCTGC	557	OL655414
*28S*	Forward ACGTCGTTGTCGATGTCC Reverse CAAGCCTTCACTTTCGTT	212	OL672488
*GAPDH*	Forward GACCACTGTCCACGCTAC Reverse GATGACACGGCTGGAGTA	451	OL675413
*EF1α*	Forward CTTCTCGCCTTCACCCTT Reverse GGCGAATCTACCCAGAGG	864	OL690518
*RPL4*	Forward TGAGAAGAGCGAGCAAGT Reverse TTTTCCCTCAGTTTCTCG	1098	OL652885
*RPL13*	Forward ACAAGGATTGGCAAAGATT Reverse ACCCTTGAGGACCTTCTT	365	OL690517
*RPL27*	Forward GAAGAACTACGACGAGGGG Reverse TGTTCTTTCCGCTCTTGTAT	299	OL675414
*SOD*	Forward ATGGTTGCTTTGCTGAAT Reverse AGATAGCTTTGACATAGTCGG	370	OL675415

**Table 2:** Primers of 10 candidate house-keeping genes used in qRT-PCR.

Gene	Primer Sequences (5′ to 3′)	Length (bp)	Slope	R^2^	Efficiency (%)
*ACT*	F-AATTGTGCGAGACGTCAAGG	239	−3.480	0.998	93.80
	R-CGTCGCACTTCATGATGGAG				
*α-TUB*	F-CACTGGTAAAGAAGACGCGG	194	−3.241	0.999	103.49
	R-AGAGACGTTCCATCAGCAGG				
*18S*	F-CGTTTGCTGGGAAGTTGACC	199	−3.289	0.997	101.39
	R-GACACGACCGTAAACCCATC				
*28S*	F-GATTCAGTTTCGGGCACTCG	154	−3.232	0.999	103.89
	R-CTAGACCGACGCTCCATCC				
*GAPDH*	F-TGCCACCCAAAAGACTGTTG	240	−3.338	0.998	99.33
	R-ACCTTGGCTTTGATCGCATC				
*EF1α*	F-TGTCAAGCAGCTGATCGTTG	164	−3.286	0.999	101.52
	R-CTCCGTGCCATCCAGAAATG				
*RPL4*	F-GGTCTGACGTGCTCAAGGTA	183	−3.452	0.991	94.84
	R-GCAGGTTCAGCTTGTCAACA				
*RPL13*	F-AACCAACCCGCTAGAAGACA	97	−3.294	0.999	101.18
	R-CCACAGGTCTCAATGGTCCA				
*RPL27*	F-TGAAGAACTACGACGAGGG	199	−3.384	0.992	97.47
	R-TCGAAGCTGAAGTCTACGGA				
*SOD*	F-CAACCTGTCTCCCTGCAAAA	159	−3.328	0.998	99.75
	R-TTCGCCAACTTGTTGTAGCC				

**Table 3:** Expression stability of the candidate reference genes under different experimental conditions.

Conditions	CRGs *	geNorm		Normfinder		BestKeeper		ΔCt	
		Stability	Rank	Stability	Rank	Stability	Rank	Stability	Rank
Developmental stages	*ACT*	1.542	9	3.751	10	2.987	10	3.819	10
	*EF*	0.208	1	0.374	2	0.289	3	1.065	1
	*18S*	0.973	8	1.214	9	1.097	9	1.728	9
	*28S*	0.429	2	0.759	7	0.284	2	1.298	6
	*SOD*	0.769	6	0.740	5	0.617	6	1.351	7
	*GAPDH*	0.721	5	0.951	8	0.878	8	1.362	8
	*α-TUB*	0.551	3	0.753	6	0.545	4	1.240	4
	*RPL4*	0.818	7	0.573	4	0.699	7	1.278	5
	*RPL13*	0.208	1	0.294	1	0.141	1	1.069	2
	*RPL27*	0.650	4	0.560	3	0.609	5	1.211	3
Larvae tissues	*ACT*	0.799	7	1.150	9	0.696	8	1.344	9
	*EF*	0.338	1	0.007	1	0.200	1	0.833	1
	*18S*	0.706	6	1.004	8	0.656	7	1.252	7
	*28S*	0.445	3	0.383	5	0.328	3	0.934	4
	*SOD*	1.161	9	2.131	10	2.088	10	2.217	10
	*GAPDH*	0.897	8	0.972	7	0.997	9	1.302	8
	*α-TUB*	0.607	5	0.608	6	0.493	5	1.043	6
	*RPL4*	0.389	2	0.272	2	0.373	4	0.869	3
	*RPL13*	0.338	1	0.293	3	0.279	2	0.868	2
	*RPL27*	0.517	4	0.370	4	0.542	6	0.946	5
Temparature treatment	*ACT*	0.614	3	0.759	6	1.293	8	1.137	5
	*EF*	0.485	1	0.502	3	0.934	5	0.991	2
	*18S*	0.903	7	0.928	8	0.755	3	1.322	8
	*28S*	0.808	6	0.812	7	0.377	1	1.194	7
	*SOD*	1.257	9	2.215	10	1.751	10	2.324	10
	*GAPDH*	0.990	8	1.200	9	1.599	9	1.451	9
	*α-TUB*	0.701	4	0.464	2	0.630	2	1.026	3
	*RPL4*	0.743	5	0.734	5	0.989	7	1.144	6
	*RPL13*	0.485	1	0.170	1	0.906	4	0.945	1
	*RPL27*	0.567	2	0.527	4	0.963	6	1.0.36	4

* Candidate reference genes.

## Data Availability

All data generated in association with this study have been made available in the Supplementary Materials published online with this article.

## Supplementary Materials

The following supporting information can be downloaded at: https://www.mdpi.com/article/10.3390/insects13020140/s1, Table S1: The overall threshold cycle (Ct) values under different experimental conditions.
