# Supplementary material for: Reference Genes for Expression Analyses by qRT-PCR in Phthorimaea operculella (Lepidoptera: Gelechiidae)
Source: Insects. 2022 Jan 28;13(2):140. doi: 10.3390/insects13020140 (PMC8879603; doi:10.3390/insects13020140)
Supplement: Supplementary file 1 [file insects-13-00140-s001.zip › insects-1525072-supplementary.pdf]

Table S1. The overall threshold cycle (Ct) values under different experimental conditions.

Conditions / CRGs*	ACT	α-TUB	18S	28S	GAPDH	EF1α	RPL4	RPL13	RPL27	SOD
Developmental stages	15.15087	20.33377	17.16861	15.40092	17.38231	18.50965	20.57646	19.09625	18.50842	24.48647
	15.30289	20.09002	17.3012	15.23966	17.40605	18.46893	20.61237	19.10226	18.18901	24.78455
	15.31202	20.0841	17.71218	15.39051	17.41345	18.36044	21.08964	19.15487	18.54869	24.62014
	21.36878	18.63741	17.24172	15.87268	15.40442	17.66621	18.93544	18.65678	17.46148	25.00257
	21.48412	18.94039	16.99137	15.90491	15.81388	17.68781	19.14562	18.96695	17.5783	26.45315
	21.61292	18.54665	17.18199	15.85825	15.48453	17.87426	19.63438	18.95512	17.6536	24.17344
	23.55853	18.74717	17.09608	15.01707	18.34377	18.35731	21.1226	19.23779	19.12414	25.91982
	23.74132	19.39036	17.1965	15.17968	17.90554	18.41681	20.8432	19.01916	19.97578	25.5086
	24.13051	19.21931	16.96727	15.00447	17.65397	18.59694	20.85534	19.31716	19.41252	25.94429
	24.2061	19.83245	19.8932	15.08853	16.32456	18.03046	21.56549	18.83839	18.21029	24.46144
	24.20983	19.82515	20.19222	15.13793	16.35854	17.87707	21.39958	18.85353	17.95341	24.56554
	24.67519	19.8523	20.31051	15.03939	16.18793	18.11147	21.65458	18.9698	17.98153	24.45445
Larvae tissues	21.41064	19.85981	16.07345	13.81916	16.75419	17.03744	19.87946	17.83532	17.98149	30.0373
	21.53244	19.65627	15.81387	13.96609	16.60983	16.93103	19.99187	17.85547	17.84878	30.17906
	21.73213	19.47741	16.54719	13.57948	16.7511	16.90399	20.09921	18.37256	17.73967	29.93597
	21.79643	18.50743	16.6471	13.96252	15.98161	17.10015	19.84644	17.84991	17.36133	24.97837
	21.85752	18.18722	17.4707	13.67054	17.10892	17.13782	20.33724	18.10657	18.55532	24.50267
	22.10178	19.14225	15.17896	14.34535	19.26302	17.25594	20.80175	18.20087	18.24593	29.1653
	22.14884	18.19736	16.81453	13.97055	16.2025	16.77641	20.14889	18.25978	17.98429	24.64478
	22.31396	19.0691	15.31338	14.25178	19.29265	17.20875	20.35391	18.16135	18.79525	29.06959
	22.3976	18.73846	15.87814	14.22464	18.37025	17.38631	21.05984	19.00373	18.66069	28.87456
	22.39895	19.11584	15.40792	14.27232	19.49422	17.1488	21.35748	18.31537	19.41829	29.68227
	22.4086	18.59349	15.54262	14.30326	17.78681	17.29609	20.89396	19.005	18.41718	28.69848
	22.43607	18.62572	15.68894	14.2478	17.69932	17.28296	21.08849	19.04397	18.8933	28.42809
	22.77226	17.78307	14.43568	14.76648	16.52638	17.06837	20.4276	17.84909	17.82487	29.04099
	22.8289	17.72235	14.62044	14.88807	16.17378	16.88747	19.932	17.78518	17.34085	28.66541
	22.96449	17.96292	14.57266	14.7814	16.66541	17.20266	20.72603	18.04869	17.33467	28.75112
	23.57818	19.42972	16.1397	13.98654	16.21541	16.74384	21.02248	17.99557	17.01243	24.54971
	23.59295	18.64676	15.01076	13.58709	15.51985	16.82157	20.2374	18.18713	17.63543	25.32356
	23.72076	19.37824	16.62145	14.05392	15.913	16.50493	20.47548	17.98661	17.0797	24.60013
	23.87847	19.15899	15.71055	14.01572	16.41601	16.60554	20.51548	18.08569	17.2835	25.01039
	23.88928	18.54342	14.90475	13.34675	15.65294	16.78447	19.79489	18.41511	17.8376	25.62803
	23.96067	18.78806	14.96591	13.43983	15.25042	17.04814	20.39293	18.28329	17.02606	25.29828
Temparature treatment	21.74472	18.3006	15.89296	14.566	16.0305	16.219	18.489	17.451	17.049	28.70632
	21.78199	18.50234	15.72709	14.459	16.16311	16.080	18.344	17.533	16.686	28.67042
	21.85881	18.18163	16.28361	13.898	16.53065	15.603	18.024	17.431	18.266	29.620
	21.87711	18.32175	15.72119	14.598	16.12239	16.186	18.752	17.500	16.914	29.1929
	22.00956	18.74751	14.46755	14.482	16.19503	16.874	21.045	17.770	17.980	30.5146
	22.15675	18.71495	14.48961	14.479	16.73127	16.048	20.687	17.356	17.182	29.87407
	22.29359	18.75954	14.25513	14.506	16.1531	16.013	20.559	17.787	17.201	30.25628
	22.37567	19.09118	15.72933	13.584	16.62312	15.837	18.268	17.171	17.926	30.124
	22.52571	18.17198	15.97678	13.930	17.19316	15.414	18.248	17.456	17.839	30.585

22.56231	18.64645	15.11902	14.769	17.24114	17.463	19.661	18.215	17.686	24.99375
22.67279	18.72953	15.48826	14.984	17.34208	17.427	19.979	18.053	17.728	25.37918
22.73264	18.297	15.17468	14.815	16.70056	16.928	19.612	18.180	17.628	25.42645
23.06645	17.77577	14.05952	14.442	17.28865	16.993	19.429	17.883	18.711	27.77227
23.2654	19.50208	14.72528	13.792	20.58568	16.741	19.607	18.264	17.980	26.97127
23.46457	18.09946	13.82993	14.034	16.93091	16.471	19.955	18.364	18.623	28.08049
23.61879	19.07186	14.53969	13.825	20.10557	16.790	19.271	17.994	18.077	26.79676
23.7222	18.15919	14.23979	14.095	17.10019	17.009	19.772	18.202	18.400	28.25869
24.21464	19.22025	14.64372	13.666	20.20391	16.886	19.556	18.132	17.646	26.9196
24.61296	20.03484	15.65906	15.210	19.05597	18.275	21.267	19.035	19.981	28.37941
24.62598	20.13625	15.68995	15.149	19.19447	18.231	21.387	19.104	19.290	28.07682
24.77825	19.95197	15.68042	15.146	19.20584	17.748	21.560	19.147	19.575	28.09458
25.71525	19.77539	14.95142	14.569	19.4011	18.448	21.173	19.809	20.073	27.34994
25.76457	19.71999	14.97772	14.560	19.68906	18.785	21.186	19.770	20.308	27.36473
25.78468	19.78948	17.75482	14.711	20.5836	18.706	21.356	20.793	19.695	33.26899
25.83033	19.59586	15.06826	14.781	19.98199	18.979	21.048	19.447	19.945	27.3571
25.91783	19.765	17.3434	15.194	20.40183	18.859	21.194	20.560	19.991	33.64865
25.94387	19.76303	16.64415	14.762	20.71656	18.539	21.156	21.058	19.745	33.74284

*Candidate reference genes
